# Differential Responsiveness of the Platelet Biomarkers, Systemic CD40 Ligand, CD62P, and Platelet-Derived Growth Factor-BB, to Virally-Suppressive Antiretroviral Therapy

**DOI:** 10.3389/fimmu.2020.594110

**Published:** 2021-01-29

**Authors:** Helen C. Steel, W. D. Francois Venter, Annette J. Theron, Ronald Anderson, Charles Feldman, Natasha Arulappan, Theresa M. Rossouw

**Affiliations:** ^1^ Department of Immunology, University of Pretoria, Pretoria, South Africa; ^2^ Ezintsha, Faculty of Health Sciences, University of the Witwatersrand, Johannesburg, South Africa; ^3^ Department of Internal Medicine, Faculty of Health Sciences, University of the Witwatersrand, Johannesburg, South Africa; ^4^ Wits Reproductive Health and HIV Institute, University of the Witwatersrand, Johannesburg, South Africa

**Keywords:** antiretroviral therapy, biomarkers, non-AIDS-defining disorders, human immunodeficiency virus, platelets, platelet-derived growth factor BB, soluble CD40 ligand, soluble CD62P

## Abstract

Systemic biomarkers of inflammation, including cytokines and chemokines, are potentially useful in the management of both HIV infection and non-AIDS-defining disorders. However, relatively little is known about the utility of measurement of circulating biomarkers of platelet activation as a strategy to monitor the efficacy of combination antiretroviral therapy (cART), as well as the persistence of systemic inflammation following virally-suppressive therapy in HIV-infected persons. These issues have been addressed in the current study to which a cohort consisting of 199 HIV-infected participants was recruited, 100 of whom were cART-naïve and the remainder cART-treated and virally-suppressed. Fifteen healthy control participants were included for comparison. The study focused on the effects of cART on the responsiveness of three biomarkers of platelet activation, specifically soluble CD40 ligand (sCD40L), sCD62P (P-selectin), and platelet-derived growth factor-BB (PDGF-BB), measured using multiplex suspension bead array technology. Most prominently sCD40L in particular, as well as sCD62P, were significantly elevated in the cART-naïve group relative to both the cART-treated and healthy control groups. However, levels of PDGF-BB were of comparable magnitude in both the cART-naïve and –treated groups, and significantly higher than those of the control group. Although remaining somewhat higher in the virally-suppressed group relative to healthy control participants, these findings identify sCD40L, in particular, as a potential biomarker of successful cART, while PDGF-BB may be indicative of persistent low-level antigenemia.

## Introduction

It is well recognized that activated platelets play a fundamental role not only in thrombosis, but also in inflammation, *via* release of prostanoids, especially thromboxane A_2_, as well as expressed and granule-packaged, pre-formed, pro-inflammatory proteins such as the adhesion receptor, CD40 ligand (CD40L) (1, 2). The soluble, secreted form of CD40L (sCD40L) is a type II membrane glycoprotein of the tumor necrosis factor (TNF) family that is expressed as a trimer on activated T cells, B cells, monocytes, macrophages, and endothelial cells, as well as platelets, contributing to atherothrombosis *via* augmentation of intravascular platelet activation and formation of platelet-leukocyte aggregates (3, 4). Notably, HIV-infected individuals often have decreased platelet counts in the setting of increased platelet activation. Although cART has been shown to effectively increase platelet counts following 3 months of treatment, platelet activation persists, possibly because of microbial translocation, resulting in sustained exposure of these cells to microbial components in the circulation (5–9). In this setting, platelet activation is likely to be the leading cause of elevated levels of circulating sCD40L detected during chronic HIV infection (1). It is noteworthy that sCD40L possesses several pro-inflammatory/prothrombotic interactions with neutrophils and macrophages that have been linked to the etiology of acute and chronic cardiovascular disorders (10–13). In this context, many of the long-term complications in HIV-infected individuals, such as ischemic thrombosis, which is one of the most common causes of death among virally-suppressed persons (14), have been associated with platelet activation (15).

Activated platelets exhibit upregulated expression of another adhesion molecule, *viz.* CD62P (P-selectin), a glycoprotein also located in α-granules. CD62P binds to its receptor, P-selectin glycoprotein ligand-1 (PSGL-1), resulting in increased adhesion of platelets to cell surfaces, promoting formation of platelet-monocyte aggregates and pro-coagulant activity (5, 16–18). As with sCD40L, sCD62P has been reported to possess pro-inflammatory/prothrombotic activities that are mediated *via* neutrophil adhesion to vascular endothelium (19) and neutrophil extracellular trap formation (20).

Platelet-derived growth factor (PDGF), which is stored in and secreted together with sCD40L and sCD62P from platelet α-granules, is expressed on the platelet surface during blood clotting, or when platelets adhere at sites of blood vessel injury. PDGF, which consists of five isoforms, is involved in cell growth and proliferation, as well as angiogenesis and arteriogenesis. It is expressed on all cell types within the atherosclerotic arterial wall, as well as on infiltrating inflammatory cells during cardiovascular disease (CVD) (21).

Loelius et al. have suggested that the combination of cigarette smoking and cART causes augmentative activation of platelets, thereby intensifying chronic systemic inflammation and increasing the risk for development of CVD (22). In this context, it is noteworthy that considerable reductions in the incidence of both CVD and coronary heart disease (CHD) have been reported in HIV-infected individuals who have managed to stop smoking (23).

The current study was motivated by our previous work on systemic biomarkers of inflammation in cART-treated and -untreated participants, which, with the exception of the chemokine, Regulated on Activation, Normal T Cell Expressed and Secreted (RANTES), did not include biomarkers of platelet origin (24). Our current study was therefore undertaken with the primary objective of identifying the effects of cART on the plasma concentrations of sCD40L, sCD62P, and PDGF-BB in ART-naïve and virally-suppressed HIV-1-infected persons.

## Methods

### Study Population

Adult (≥18 years) HIV-infected participants attending the Antiretroviral Clinic at the Charlotte Maxeke Johannesburg Academic Hospital in Johannesburg, South Africa, were recruited as described previously (24). Of these, 100 were cART-naïve, while the remaining 99 were cART-treated and virally-suppressed. Participants received routine care as prescribed by the South African Department of Health HIV Treatment Guidelines (25). This guideline follows a public health approach; accordingly, only CD4 T-cell count and serum creatinine testing are routinely performed before the initiation of treatment. Full blood count is only performed in patients who are started on zidovudine or are clinically anemic, while HIV viral load (VL) is only performed after 6 months of treatment. Fasting cholesterol testing is reserved for patients started on a lopinavir-based regimen. For this study, CD4 T-cell counts were obtained from routine programmatic data. HIV VL was available for participants on treatment and was retrospectively performed by the National Health Laboratory Service (NHLS) of South Africa on stored samples from treatment-naïve participants, using the same platform: Abbott M200sp. Fifteen healthy, HIV-uninfected participants of the same socio-demographic groups were included to serve as controls. Ethics clearance for the study was granted by the Faculty of Health Sciences Research Ethics Committee, University of Pretoria, Pretoria, South Africa, and the Human Research Ethics Committee of the University of the Witwatersrand, Johannesburg, South Africa (Ethics reference numbers 94/2013 and M130383, respectively). Whole blood samples were collected in EDTA vacutainers and the plasma separated and frozen at minus 80°C.

### Platelet Biomarkers

We have previously reported on systemic chemokine/cytokine profiles, as well as biomarkers of neutrophil activation, in the same cohort of HIV-infected participants described in the current study (24). One of these biomarkers was the chemokine, RANTES, which is stored in platelet α-granules, the levels of which were significantly elevated in cART-naïve participants and decreased markedly following virally-suppressive therapy (24, 26). This study provided the basis for the current follow-up study in which stored plasma samples were re-analyzed to measure platelet activation markers. Soluble CD40L, PDGF-BB, and sCD62P were quantified in plasma using the Bio-Plex suspension array platform together with Milliplex^®^ Map assay kits (EMD Millipore Corporation, Billerica, MA, USA) according to the manufacturer’s instructions and the results expressed as picograms (pg)/ml plasma in the case of sCD40L and PDGF, and nanograms (ng)/ml in the case of sCD62P.

### Tobacco Usage Status

Tobacco usage was determined by measuring plasma cotinine, an objective measure of tobacco use. For this purpose, an ELISA procedure (Calbiotech, Spring Valley, CA, USA) was used with levels of ≥15 ng/ml taken as being positive for tobacco use (27).

### Statistical Analysis

Distribution-free statistics were employed as data were not normally distributed. Descriptive results are presented as proportions and frequencies, as well as medians and interquartile ranges (IQR). Tests of association were performed by Chi-square or Fisher’s exact tests as appropriate, or by the Kruskal-Wallis test, used for comparing two or more independent samples. Univariate and multivariable analyses were performed by means of nonparametric local–constant kernel regression since it makes no assumptions about the relationship between the outcome variable and the covariates. For this analysis, VL was dichotomized into VL ≤40 and >40 copies/ml. Analysis was performed in Stata 16 (StataCorp. 2019, College Station, Texas, USA). P values below 0.05 were considered statistically significant.

## Results

Two hundred and fourteen participants were recruited to the study: 199 HIV-infected participants and 15 HIV-uninfected controls with the same socio-demographic characteristics. All cART-treated participants were on standard first-line treatment consisting of tenofovir disoproxil fumarate (TDF), emtricitabine (FTC), and efavirenz (EFV). Participants had started on cART a median of 14 (IQR 14–19) months earlier, and all had an undetectable viral load (VL) (≤40 copies/ml) at the time of the study. The 100 cART-naïve participants were about to commence cART at the same clinic and had a median CD4 T-cell count of 209 cells/mm^3^ (IQR 115-305) and VL of 28,429.5 copies/ml (IQR 5,432–111,373). Of all participants, 138 (69.4%) admitted to having ever used tobacco and 70 (30.6%) were current tobacco users as determined by cotinine levels. None of the controls used tobacco to allow for the assessment of the impact of both HIV and tobacco use. No gender differences were observed between the groups. **Table 1** shows the demographic, clinical and biomarker results and comparisons between the three groups: cART-naïve; virally-suppressed; and HIV-uninfected controls. As expected, the median CD4 T-cell count was significantly higher and the HIV VL significantly lower in the cART-treated group relative to the cART-naïve group.

**Table 1:** Comparison of the demographic, clinical, and biomarker results between the three groups.

	Group 1 (cART-naïve)n=100	Group 2 (Suppressed)n=99	1 vs 2P-value	Control n=15	1 vs ControlP-value	2 vs ControlP-value
Age (years)	33(29–37)	34(31–41)	0.0518	33(28–36)	0.0506	0.2127
Gender(female)	64(64%)	54(54.5%)	0.175	9(60%)	0.764	0.692
CD4 count* (cells/μl)	209(115–305)	348(240–464)	**0.0001**	N/A		
HIV VL** (copies/ml)	28,429.5(5,432–111,373)	40(40–40)	**<0.0001**	N/A		
sCD40L(pg/ml)	457.58(178.9–1441.68)	131.02(72.98–287.18)	**<0.0001**	48.02(2.39–229.22)	**<0.0001**	**0.0258**
sCD62P(ng/ml)	226.86(179.93–354.46)	209.51(182.08–231.32)	**0.005**	194.51(177.77–211.08)	**0.0073**	0.1481
PDGF-BB(pg/ml)	9460.55(3228.73–24300.08)	8699.6(6132.38–11626.2)	0.0843	4456.81(41.39–10118.03)	**0.0028**	**0.0194**

All results are shown as median values with interquartile ranges (IQRs) except for gender, displayed as n (%).

*CD4 counts were not available for 1 participant in each of the HIV-infected groups.

**VL, viral load. Available for 85 participants in group 1 and all participants in group 2. Bold font indicates statistically significant p-values.

**Figure 1:**
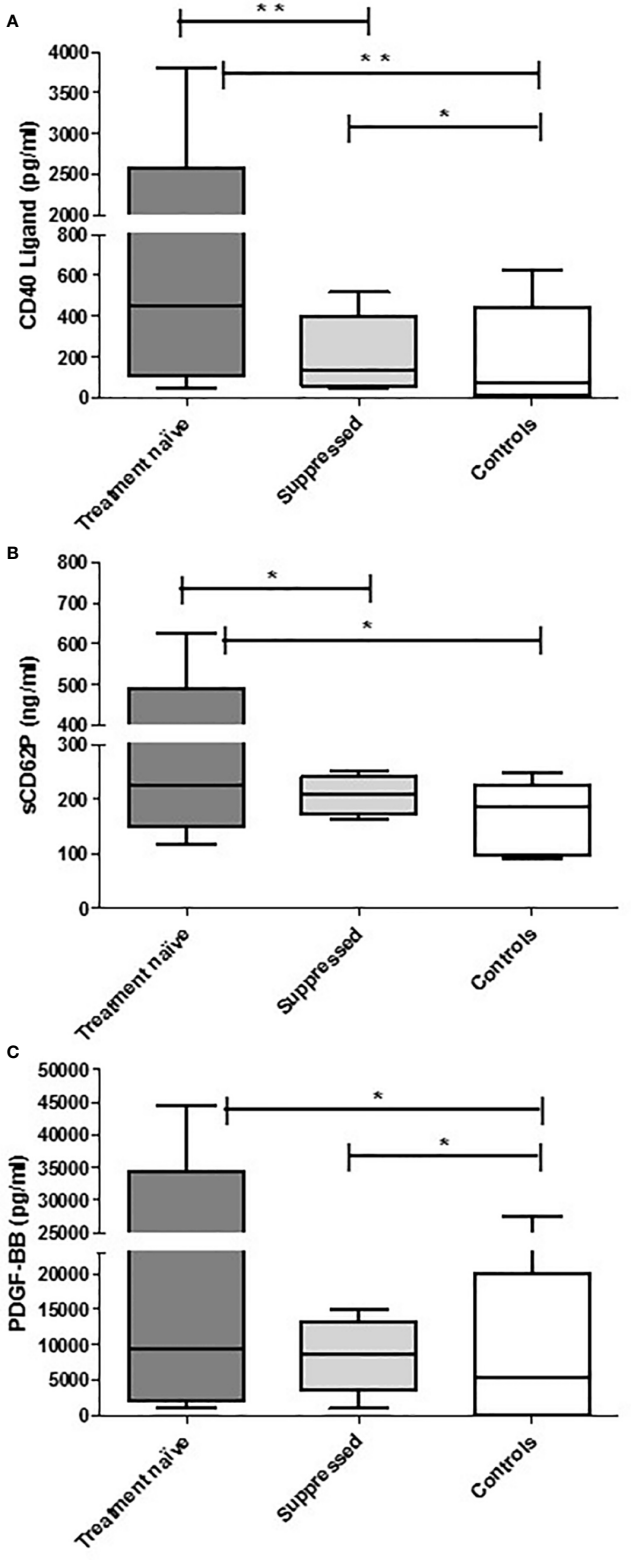
The concentrations of CD40 ligand **(A)**, CD62P **(B)**, and PDGF-BB **(C)** in the plasma of cART-naïve (n=100), virally suppressed (n=99), and control (n=15) participants. The results are presented as the median values with interquartile ranges. *0.05 < *P* ≥ 0.0001. **P < 0.0001.

With respect to differences in the levels of the test biomarkers between groups, the following were noted: i) levels of sCD40L and sCD62P were significantly higher in the cART-naïve group than in the suppressed group; ii) levels of sCD40L were higher in both of the HIV-infected groups versus controls; iii) levels of sCD62P were significantly higher in the cART naïve group when compared with controls and while higher, no statistically significant difference was observed between the suppressed group and the controls. In contrast, there was no difference in PDGF-BB levels between the two HIV-infected groups; however, the levels of this biomarker were significantly higher in both HIV-infected groups relative to the control group. These results are depicted in **Table 1** as well as in **Figures 1A–C**.

To explore possible differences in the effect of cART versus that of tobacco usage on systemic concentrations of the three platelet biomarkers, the HIV-infected groups were subdivided according to cART status and tobacco use into four sub-groups, namely: cART-naïve users and non-users of tobacco and cART-treated users and non-users of tobacco. **Table 2** demonstrates differences between the cART-treated and -naïve groups with respect to CD4 T-cell counts, cotinine levels and concentrations of the test biomarkers between these sub-groups. As expected, plasma cotinine levels were significantly higher and of comparable magnitude in the two sub-groups of tobacco users. Likewise, CD4 T-cell counts were significantly lower and also of comparable magnitude in the two cART-naïve sub-groups relative to those of the two cART treated sub-groups and were unaffected by tobacco usage.

**Table 2:** Analysis according to ART status and tobacco use.

	Group 1 cART-naïve Tobacco user n = 32	Group 2 On cART Tobacco user n = 23	Group 3 cART-naïve Non-user n = 68	Group 4 On cART Non-user n = 76	1 vs 2 P-value	1 vs 3 P-value	1 vs 4 P-value	2 vs 3 P-value	2 vs 4 P-value	3 vs 4 P-value
CD4 count* (cells/μl)	211(137.5–305)	344(315–466)	209(106–307)	351.5(237.5–454.5)	**0.0001**	0.413	**0.0001**	**0.0001**	0.267	**0.0001**
HIV VL** (copies/ml)	24,251(6,081–42,954)	40(40–40)	32,162(5,432–145,630)	40(40–40)	**<0.0001**	0.4799	**<0.0001**	**<0.0001**	0.500	**<0.0001**
Cotinine(ng/ml)	100(100–100)	100(67.3–100)	0(0–0)	0(0–0)	0.363	**0.0001**	**0.0001**	**0.0001**	**0.0001**	0.361
sCD40L(pg/ml)	678.78(193.81–1458.37)	105.47(63.75–265.13)	412.55(174.48–1360.9)	143.57(74.39–289.89)	**0.0003**	0.3320	**0.0001**	**0.0002**	0.2737	**<0.0001**
sCD62P(ng/ml)	241.92(179.93–472.3)	209.51(182.98–226.86)	223.55(180.38–296.16)	209.51(180.58–236.64)	**0.0208**	0.2018	**0.0088**	0.0585	0.4057	**0.0271**
PDGF-BB (pg/ml)	9531.28(3620.74–30988.8)	8087.92(7271–9815.04)	9460.55(3095.84–20669.42)	9004.54 (5400.24–11876.42)	0.1301	0.1928	0.0529	0.3068	0.4449	0.1769

All results are shown as the median values with interquartile ranges (IQRs).

*CD4 counts were not available for 1 participant in Group 2 and 1 participant in Group 3. **VL, viral load. Available for 85 participants in groups 1 and 3 and all participants in groups 2 and 4. Bold font indicates statistically significant p-values.

Plasma levels of sCD40L mimicked the differences observed between the entire cART-naïve and -treated groups with higher levels observed in cART-naïve participants, regardless of tobacco use. Soluble CD62P demonstrated a similar pattern, although the difference between participants on cART who used tobacco and cART-naïve non-users narrowly missed significance. No difference was observed between any of the sub-groups for PDGF-BB. Combined ART-naïve participants, regardless of tobacco use, differed from the HIV-uninfected controls with respect to all three parameters tested. Virally-suppressed participants, regardless of tobacco use, had higher levels of PDGF-BB than controls, and non-tobacco-users also had higher levels of sCD40L than controls (**Supplementary Table 1**). When all HIV-infected participants were grouped according to tobacco use status, there was no difference between the HIV-infected groups in any of the biomarkers tested, while levels of all three biomarkers remained significantly higher than controls (**Supplementary Table 2**).

The results of the univariate analysis of the three biomarkers with one another and all the available clinical variables are shown in **Table 3**. All three biomarkers, sCD40L, sCD62P, and PDGF-BB, were significantly associated with one another (P<0.0001). In addition, sCD40L was also significantly associated with VL (p=0.001), an association which persisted in the multivariable analysis (p=0.012) (**Table 4**). In addition, PDGF-BB was weakly, but significantly, associated with cotinine (p=0.045) but this association disappeared in the multivariable model (p=0.113) and only its associations with sCD40L (p<0.0001) and sCD62P (p=0.003) remained significant.

**Table 3:** Univariate analysis.

	sCD40L	sCD62P	PDGF-BB
**sCD40L**	–	**<0.0001**	**<0.0001**
**sCD62P**	**<0.0001**	–	**<0.0001**
**PDGF-BB**	**<0.0001**	**<0.0001**	–
**Age**	0.616	0.311	0.750
**Gender**	0.123	0.875	0.262
**CD4**	0.061	0.133	0.120
**VL**	**0.001**	0.130	0.304
**Cotinine**	0.445	0.452	**0.045**

Bold font indicates statistically significant p-values.

**Table 4:** Multivariable analysis of sCD40L.

	Mean	Effect			
PDGF-BB	4607.58	19118.47			
CD62P	216.4203	898.0038			
vl_cat	.5	.5			
Local-linear regression	Number of obs	= 170			
Continuous kernel:	epanechnikov	E(Kernel obs)	= 170		
Discrete kernel:	liracine	R-squared	= 0.8263		
Bandwidth:	cross validation				
Observed	Bootstrap	Percentile			
CD40L	Estimate	Std. Err.	z	P>z	[95% Conf. Interval]
Mean					
CD40L	525.5692	81.48458	6.45	**0.000**	334.117605.3802
Effect					
PDGF-BB	.0415206	.0111245	3.73	**0.000**	.0198329 .0602123
CD62P	3.566333	1.493815	2.39	**0.017**	.1424808 5.489959
vl_cat					
(2 vs 1)	181.8935	72.46938	2.51	**0.012**	83.81196 333.6938

Effect estimates are averages of derivatives for continuous covariates and averages of contrasts for factor covariates. Bold font indicates statistically significant p-values.

## Discussion

Our findings demonstrate that the concentrations of the three test biomarkers associated with platelet activation, sCD40L, sCD62P, and PDGF-BB, all of which are located in the α-granules (28), were significantly elevated in the plasma of cART-naïve, HIV-1-infected participants. Following administration of cART, plasma concentrations of two of the test biomarkers, *viz.* the potent inflammatory mediators, sCD40L and sCD62P, but not those of PDGF-BB, were found to be significantly lower in the cART-treated, virally-suppressed group of participants. Nevertheless, both sCD40L and sCD62P remained elevated when comparing the virally-suppressed and control groups, although only the difference in levels of sCD40L attained statistical significance. It is noteworthy that only sCD40L was associated with VL in univariate and multivariate analysis.

These findings, which are consistent with a beneficial anti-inflammatory effect of cART, are generally in keeping with an earlier study, which reported a reduction in systemic levels of platelet markers measured prior to and 12 weeks after initiation of cART in a small group (n=25) of HIV-infected participants, (29). Our observation of a sustained elevation in sCD40L, particularly in the virally-suppressed group relative to the control group is, however, indicative of persistent, low-level platelet activation, even in the setting of administration of successful cART (16, 30). This contention is in keeping with the findings of Real *et al.* who recently reported that platelets from patients on virally-suppressive cART harbor replication-competent HIV that is associated with poor immune restoration (31). Apart from acting as a viral reservoir and potential source of persistent HIV dissemination, it is intriguing, albeit speculative, that persistent viral replication within platelets may be the cause of the persistent platelet activation observed in the current study.

In addition to HIV, platelets also harbor other single stranded RNA viruses, most notably influenza virus and SARS-CoV-2 that also promote potentially harmful systemic inflammatory responses, as well as viral dissemination and infection of CD4 T-cells and macrophages (32–34). Although the types of platelet/virus pro-adhesive receptor/ligand interactions vary somewhat (33, 35), the common and possibly the most prominent of these involves recognition of viral single-stranded RNA by platelet Toll-like receptor (TLR) 7 (32, 36, 37).

Following implementation of virally-suppressive cART, the plasma concentrations of PDGF-BB, a biomarker which has received relatively little attention in the setting of HIV-1 infection, remained almost unchanged (8% decrease, not significantly different from those of the cART-naïve group). This observation suggests that platelets may not be the major cellular source of plasma PDGF-BB in those infected with HIV-1. Unlike sCD40L and sCD62P, which are highly concentrated in platelets (28, 38), PDGF-BB is also produced by various cell types, including monocytes/macrophages, endothelial cells, fibroblasts, smooth muscle cells, and neuronal cells (39) and is the only one of the five isoforms of PDGF which interacts with all three PDGF receptor sub-types (40). Given the diverse cellular origins of PDGF-BB, particularly its production by monocytes/macrophages and endothelial cells, which like platelets are reported to serve as residual reservoirs of HIV (41, 42), persistently elevated levels of PDGF-BB may be indicative of persistent low-level viral antigenemia. In this context, a recent study undertaken in HIV-1-infected males who were virally suppressed for almost two decades, is particularly noteworthy (43). This study was focused, amongst others, on alterations in the plasma concentrations of a large number of plasma proteins (n=92), encompassing cytokines, chemokines, growth factors, and cell surface markers, measured over the entire time course of viral suppression (26). Of the biomarkers included in the proteomic profile, only two remained significantly elevated following the prolonged period of viral suppression. These were sKLRD1 (expressed on natural killer cells) and PDGF-B (subunit B) (43).

Interestingly, PDGF-BB has also been implicated in the pathogenesis of coronary atherosclerosis (44), a common non-AIDS-defining disorder, with a prevalence twice that of HIV-uninfected persons (45). In this setting, disease pathogenesis involves PDGF-BB-mediated induction of smooth muscle cell proliferation together with phenotypic changes to these cells, promoting migration and formation of neointima (46, 47).

With respect to tobacco usage, this was not associated with significant elevations in any of the test biomarkers of platelet activation in our cohort of HIV-infected participants. In our opinion, this was a surprising finding given the following: i) previous reports that tobacco usage *per se* results in platelet activation that is associated with significant elevations in circulating sCD40L (48, 49); and ii) exposure to nicotine in an animal model of experimental atherogenesis resulted in expression of the gene encoding PDGF-B in aortic tissue (50). In this particular cohort of HIV-infected participants, it therefore appears that any potential effect of tobacco usage on platelet activation may have been overshadowed by the dominant influence of HIV infection *per se*.

We do concede that our study is somewhat limited by its cross-sectional nature and relatively short duration of cART administration, which preclude assessment of possible associations between the biomarkers and clinical outcomes. Nevertheless, the findings may provide a basis for future clinical studies focused on these issues. In addition, our findings confirm and extend earlier studies, which indicate that biomarkers are of potential importance in complementing routine VL monitoring, which is unable to detect very low-level viremia and ongoing viral replication outside of the blood compartment. Biomarkers may also have clinical utility in the context of individualized prevention and treatment, especially given the current controversy surrounding the exact place of statins and platelet-targeted therapies in the prevention of CVD in this population (51, 52). In this context, a very recent study by Falasca *et al.* is noteworthy (53). These authors, albeit in a smaller cohort of cART-treated patients (n=80) than that described in the current study, reported that circulating levels of sCD40L, but not sCD62P, were significantly elevated relative to those of a matched, control group of HIV-negative participants (n=80), and were associated with the presence of hypertension (53). This study is limited, however, by the absence of a group of cART-naïve-participants, while the large proportion of participants with co-morbid diabetes mellitus (17.5%) and arterial hypertension (40%) makes comparison with our study difficult.

## Conclusion

In conclusion, the results of the current study have demonstrated a novel, anti-inflammatory activity of cART that is associated with attenuation of the plasma concentrations of the predominantly platelet-derived, pro-inflammatory factors, sCD40L in particular, as well as sCD62P. Nevertheless, the finding that sCD40L remained significantly elevated in the virally-suppressed group, albeit at a lower level than pre-cART, may be indicative of chronic, systemic low-grade inflammation, while the observed lack of effect of cART on plasma levels of PDGF-BB may reflect the various cellular origins of this cytokine and a possible association with persistent, low-level antigenemia. Notwithstanding prioritization of strategies to eliminate latent viral reservoirs, future research should focus on identifying associations, if any, of plasma sCD40L with the presence of cardio-metabolic disease in those patients receiving virally-suppressive cART.

## Data Availability Statement

The raw data supporting the conclusions of this article will be made available by the authors, without undue reservation.

## Ethics Statement

The studies involving human participants were reviewed and approved by Faculty of Health Sciences Research Ethics Committee, University of Pretoria, Pretoria, South Africa, and the Human Research Ethics Committee of the University of the Witwatersrand, Johannesburg, South Africa (Ethics reference numbers 94/2013 and M130383, respectively). The patients/participants provided their written informed consent to participate in this study.

## Author Contributions

All authors contributed equally to conceptualize the study. WV, NA, CF, and TR were involved in the recruitment of HIV-infected participants and gave clinical oversight and input. HS, RA, and AT performed the laboratory tests. All authors contributed to the article and approved the submitted version.

## Funding

The research in this article was funded by a National Health Laboratory Service Development Grant (004 94706). The Bill and Melinda Gates Foundation, as well as the South African Department of Health and South African Medical Research Council, supported the clinical trial that contributed specimens to this study. Charles Feldman is supported by the National Research Foundation of South Africa. We would like to thank the patients and staff of the Charlotte Maxeke Johannesburg Academic Hospital.

## Conflict of Interest

The authors declare that the research was conducted in the absence of any commercial or financial relationships that could be construed as a potential conflict of interest.

## Supplementary Material

Supplementary Table 1 Tests of association between HIV-infected (treatment naïve versus virally-suppressed) tobacco users and non-users and HIV-uninfected controls.

Supplementary Table 2 Tests of association among three groups: HIV-infected tobacco users, non-users, and HIV-uninfected controls. Groups: 1 = All HIV+ smokers; 2 = All HIV+ non-smokers; 3 = HIV-uninfected controls.
