# Supplementary material for: Differential Responsiveness of the Platelet Biomarkers, Systemic CD40 Ligand, CD62P, and Platelet-Derived Growth Factor-BB, to Virally-Suppressive Antiretroviral Therapy
Source: Front Immunol. 2021 Jan 29;11:594110. doi: 10.3389/fimmu.2020.594110 (PMC7878378; doi:10.3389/fimmu.2020.594110)
Supplement: Supplementary Table 1 — Tests of association between HIV-infected (treatment naïve versus virally-suppressed) tobacco users and non-users and HIV-uninfected controls. [file Table_1.docx]

**Supplementary Table 1: Tests of association between HIV-infected (treatment naïve versus virally-suppressed) tobacco users and non-users and HIV-uninfected controls**

sCD40L


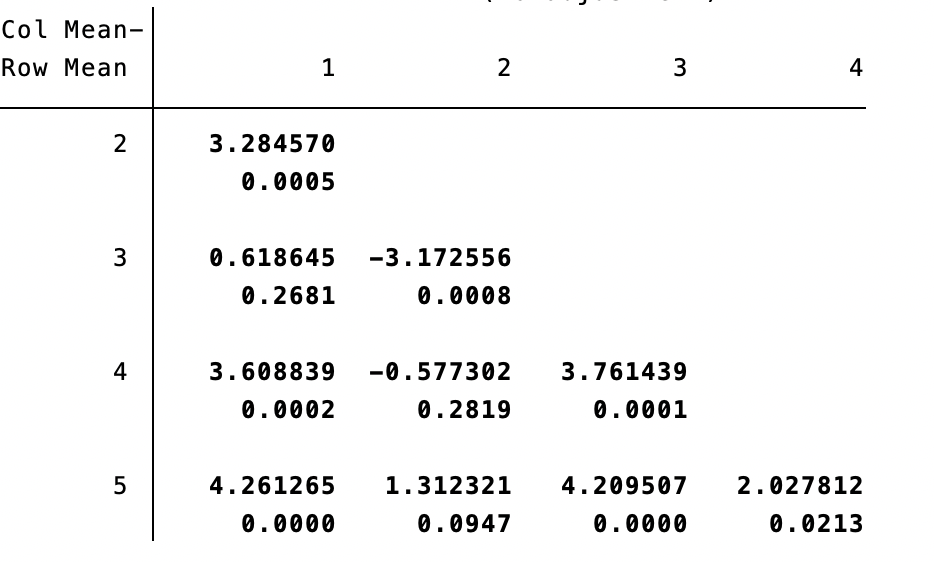


sCD62P


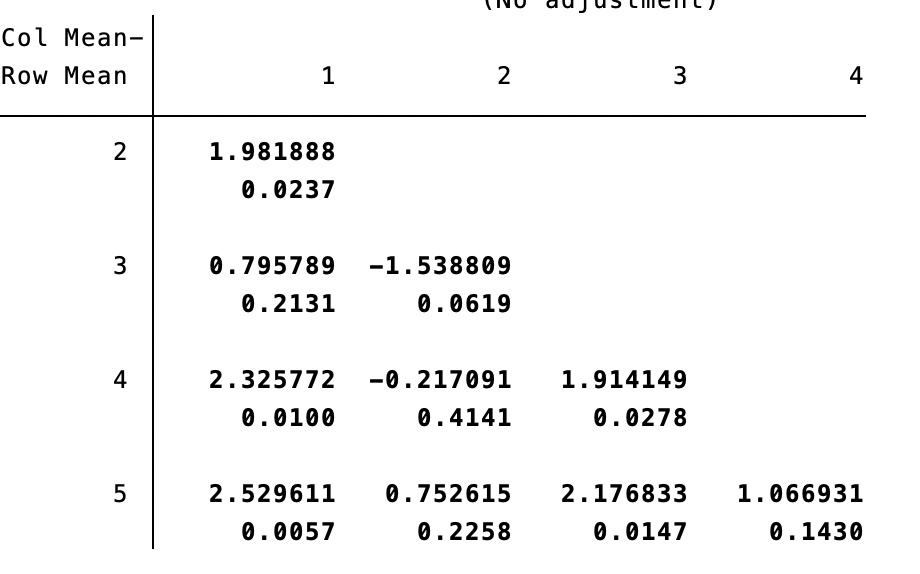


PDGF-BB

**
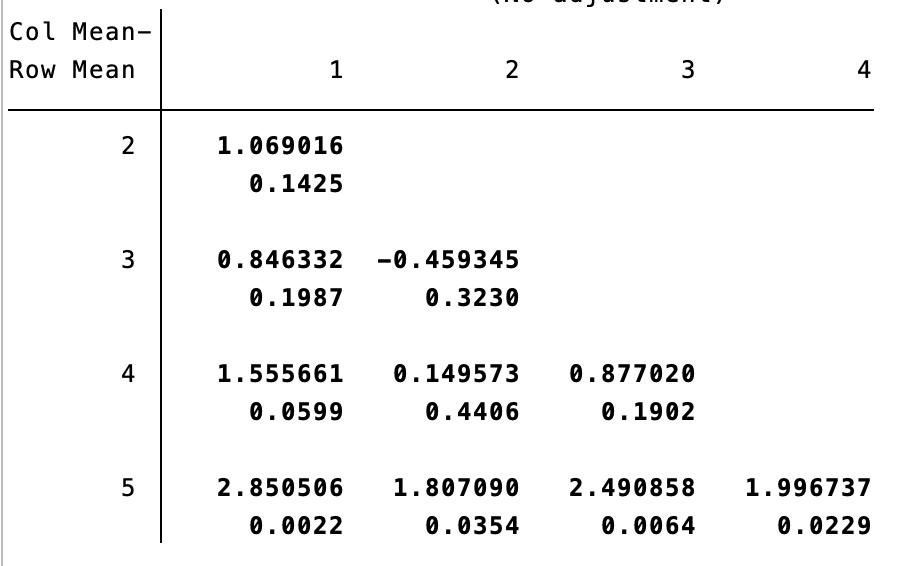
**

Groups: 1 = ART-naïve tobacco user (n=32); 2 = Virally suppressed tobacco user (n=23); 3 = ART-naïve non-tobacco user (n=68); 4 = Virally suppressed non-tobacco user (n=76); 5 = HIV-uninfected controls (n=15)
