# Supplementary material for: Differential Responsiveness of the Platelet Biomarkers, Systemic CD40 Ligand, CD62P, and Platelet-Derived Growth Factor-BB, to Virally-Suppressive Antiretroviral Therapy
Source: Front Immunol. 2021 Jan 29;11:594110. doi: 10.3389/fimmu.2020.594110 (PMC7878378; doi:10.3389/fimmu.2020.594110)
Supplement: Supplementary Table 2 — Tests of association among three groups: HIV-infected tobacco users, non-users, and HIV-uninfected controls. Groups: 1 = All HIV+ smokers; 2 = All HIV+ non-smokers; 3 = HIV-uninfected controls. [file Table_2.docx]

**Supplementary Table 2: Tests of association among three groups: HIV-infected tobacco users, non-users, and HIV-uninfected controls**

sCD40L


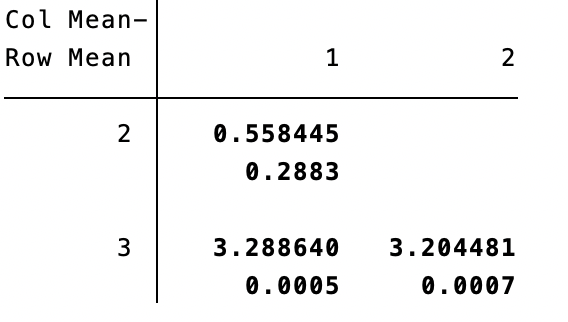


sCD62P


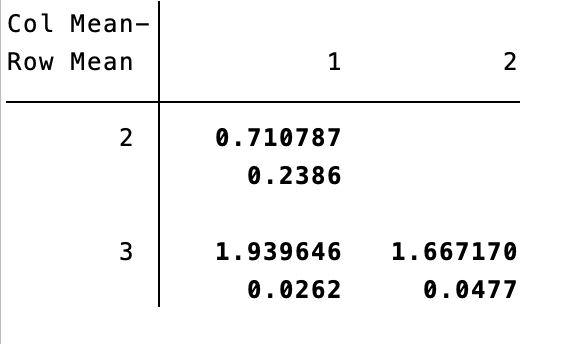


PDGF-BB


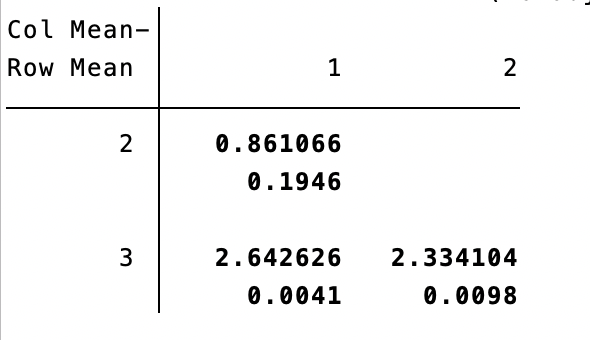


Groups: 1 = All HIV-infected smokers (n=55); 2 = All HIV-infected non-smokers (n=144); 3 = HIV-uninfected controls (n=15)
